# Memorial to the late Sir Edmund Lechmere, Bart., M.P.

**Published:** 1895-06-15

**Authors:** 


### Editor's Letter-Box.

[Our correspondents are reminded that proxility is a great bar to publication, and that brevity of style and conciseness of statement greatly facilitate early insertion.]

### MEMORIAL TO THE LATE SIR EDMUND LECHMERE, BART., M.P.

Lord Egerton of Tatton writes: There are many friends of the late Sir Edmund Lechmere, M.P., who are aware how much he interested himself in the establishment of an ophthalmic hospital at Jerusalem, under the management of the Order of the Hospital of St. John of Jerusalem in England, and who would be glad to subscribe to a fund for placing that hospital on a permanent basis as a memorial of the services rendered by him in that and other philanthropic work. This proposal, I am permitted to state, has the entire concurrence of the Grand Prior of the Order, His Royal Highness the Prince of Wales, and as the chairman of the committee appointed by His Royal Highness, I should be glad, through your columns, to invite support to the scheme.

The British Ophthalmic Hospital was established at Jerusalem in the year 1881, and the magnitude of the work may be estimated by the simple statement that there is no other hospital in Palestine dealing with the numerous diseases of the eye so prevalent in that climate, so that people come from all parts of the Holy Land to seek the restoration of sight, or to obtain relief from suffering. The report furnished by the medical officer in charge for the last three years is as follows: Applicants for admission, 6,068; in-patients, 2,379; out-patients, new cases, 25,616; out-patients, total cases, 69,379; operations, 4,823.

It is sad to record that during the last year 905 applicants were turned away because they could not be treated with any hope of success as in-patients, and there were neither beds in which to receive them nor funds to pay for their support. The hospital stands alone, among the charitable institutions of Jerusalem, in preserving an attitude of absolute impartiality among the holders of different creeds, and in admitting to its benefits, on equal terms, Christians, Jews, and Mohammedans, not only without any attempt to make proselytes, but with full security for religious freedom to them all.

The present annual subscriptions are inadequate for the maintenance of the hospital, which, even in its present state of efficiency, requires an annual income of at least £900. This appeal is to raise an amount necessary to endow the hospital, and thus, not only to maintain, but to widen the sphere and to increase the scope and usefulness of a charity which has won for itself a first place among the institutions of the Holy Land.

I am authorised to add that Katherine, Lady Lechmere, will contribute £1,000 as a donation to the fund.

Donations will, unless otherwise directed, be acknowledged in your columns, and should be sent to the Honorary Secretary, Mr. R. Gofton-Salmond, 73, Cheapside, E.C., or to the account of the hospital at the London and Westminster Bank.

